# Establishing international optimal cut-offs of waist-to-height ratio for predicting cardiometabolic risk in children and adolescents aged 6–18 years

**DOI:** 10.1186/s12916-023-03169-y

**Published:** 2023-11-15

**Authors:** Xin’nan Zong, Roya Kelishadi, Young Mi Hong, Peter Schwandt, Tandi E. Matsha, Jose G. Mill, Peter H. Whincup, Lucia Pacifico, Abel López-Bermejo, Carmelo Antonio Caserta, Carla Campos Muniz Medeiros, Anastasios Kollias, Mostafa Qorbani, Fariborz Sharifian Jazi, Gerda-Maria Haas, Rafael de Oliveira Alvim, Divanei Zaniqueli, Claudio Chiesa, Judit Bassols, Elisabetta Lucia Romeo, Danielle Franklin de Carvalho, Mônica Oliveira da Silva Simões, George S. Stergiou, Evangelos Grammatikos, Min Zhao, Costan G. Magnussen, Bo Xi

**Affiliations:** 1 Department of Epidemiology, School of Public Health, Shandong University, Jinan, China; 2 Department of Growth and Development, Capital Institute of Pediatrics, Beijing, China; 3 Child Growth and Development Research Center, Research Institute for Primordial Prevention of Non Communicable Disease, Isfahan University of Medical Sciences, Isfahan, Iran; 4 Department of Pediatrics, Ewha Womans University School of Medicine, Seoul, Korea; 5 Atherosclerosis Prevention Institute, Munich-Nuremberg, Munich, Germany; 6 Department of Biomedical Sciences, Faculty of Health & Wellness Sciences, Cape Peninsula University of Technology, Cape Town, South Africa; 7 Department of Physiological Sciences, Center of Health Sciences, Federal University of Espírito Santo, Vitória, Brazil; 8 Population Health Research Institute, St George’s, University of London, London, UK; 9 Department of Maternal and Child Health, Sapienza University of Rome, Rome, Italy; 10 Pediatric Endocrinology Research Group, Girona Biomedical Research Institute (IDIBGI), Salt, Spain; 11 Department of Pediatrics, Hospital Dr. Josep Trueta, Girona, Spain; 12 Department of Medical Sciences, University of Girona, Girona, Spain; 13 Associazione Calabrese Di Epatologia - Medicina Solidale - A.C.E. ETS, Reggio Calabria, Italy; 14 Department of Public Health, State University of Paraiba, Campina Grande, Brazil; 15 Hypertension Center STRIDE-7, School of Medicine, Third Department of Medicine, National and Kapodistrian University of Athens, Sotiria Hospital, Athens, Greece; 16 Non Communicable Research Center, Alborz University, Karaj, Iran; 17 School of Science and Technology, University of Georgia, Tbilisi, Georgia; 18 Department of Public Health, Federal University of Espirito Santo, Vitória, ES Brazil; 19 Institute of Translational Pharmacology, National Research Council, Rome, Italy; 20 Maternal-Fetal Metabolic Research Group, Girona Institute for Biomedical Research (IDIBGI), Salt, Spain; 21 Health Center of Samos, Vathi, Samos Greece; 22 Department of Nutrition and Food Hygiene, School of Public Health, Shandong University, Jinan, China; 23 Baker Heart and Diabetes Institute, Melbourne, VIC Australia; 24 Research Center of Applied and Preventive Cardiovascular Medicine, University of Turku, Turku, Finland; 25 Centre for Population Health Research, University of Turku and Turku University Hospital, Turku, Finland

**Keywords:** Waist-to-height ratio, Central obesity, Cardiovascular risk factors, Child, Adolescent

## Abstract

**Background:**

Waist-to-height ratio (WHtR) has been proposed as a simple and effective screening tool for assessing central obesity and cardiometabolic risk in both adult and pediatric populations. However, evidence suggests that the use of a uniform WHtR cut-off of 0.50 may not be universally optimal for pediatric populations globally. We aimed to determine the optimal cut-offs of WHtR in children and adolescents with increased cardiometabolic risk across different countries worldwide.

**Methods:**

We used ten population-based cross-sectional data on 24,605 children and adolescents aged 6–18 years from Brazil, China, Greece, Iran, Italy, Korea, South Africa, Spain, the UK, and the USA for establishing optimal WHtR cut-offs. We performed an external independent test (9,619 children and adolescents aged 6–18 years who came from other six countries) to validate the optimal WHtR cut-offs based on the predicting performance for at least two or three cardiometabolic risk factors.

**Results:**

Based on receiver operator characteristic curve analyses of various WHtR cut-offs to discriminate those with ≥ 2 cardiometabolic risk factors, the relatively optimal percentile cut-offs of WHtR in the normal weight subsample population in each country did not always coincide with a single fixed percentile, but varied from the 75^th^ to 95^th^ percentiles across the ten countries. However, these relatively optimal percentile values tended to cluster irrespective of sex, metabolic syndrome (MetS) criteria used, and WC measurement position. In general, using ≥ 2 cardiometabolic risk factors as the predictive outcome, the relatively optimal WHtR cut-off was around 0.50 in European and the US youths but was lower, around 0.46, in Asian, African, and South American youths. Secondary analyses that directly tested WHtR values ranging from 0.42 to 0.56 at 0.01 increments largely confirmed the results of the main analyses. In addition, the proposed cut-offs of 0.50 and 0.46 for two specific pediatric populations, respectively, showed a good performance in predicting ≥ 2 or ≥ 3 cardiometabolic risk factors in external independent test populations from six countries (Brazil, China, Germany, Italy, Korea, and the USA).

**Conclusions:**

The proposed international WHtR cut-offs are easy and useful to identify central obesity and cardiometabolic risk in children and adolescents globally, thus allowing international comparison across populations.

**Supplementary Information:**

The online version contains supplementary material available at 10.1186/s12916-023-03169-y.

## Background

Childhood and adolescent obesity, a global epidemic [1, 2], often accompanies cardiometabolic risk factors such as high blood pressure (BP), elevated triglycerides (TG), low high-density lipoprotein cholesterol (HDL-C), and high fasting blood glucose (FBG) [3]. The accumulation of abdominal fat, more so than fat in other areas of the body, has a considerable impact on metabolic variables, which often cluster, leading to conditions like metabolic syndrome (MetS).

Body mass index (BMI) is the predominant adiposity metric to assess overweight and obesity in clinical practice and epidemiological studies due to its simplicity and reliability of its input measurements of weight and height. Several international organizations, including the World Health Organization (WHO) [4] and the International Obesity Task Force (IOTF) [5] have defined pediatric overweight and obesity with age- and sex- specific BMI percentile cut-offs. However, BMI has several limitations, such as its inability to accurately assess fatness, fat distribution (including visceral fat), and the need for percentile tables due to variation by age and sex in children and adolescents. Meta-analyses indicate that waist circumference (WC), a marker of central obesity, is more strongly correlated with the presence of cardiometabolic risk factors than BMI in both adults and children [6, 7]. Despite the recent issue of region-specific international WC percentile cut-offs for defining central obesity in children and adolescents [8], WC also requires age- and sex-specific cut-offs to account for variation. Moreover, the cardiometabolic risk may differ between individuals with the same WC but different heights [9].

As a further alternative, the waist-to-height ratio (WHtR) has been proposed as a simple and effective adiposity metric to assess central obesity and cardiometabolic risk [10–12]. A WHtR value of 0.50, notably more discriminant than WC or BMI for predicting cardiometabolic disease and diabetes in adults [12–14], is widely applied to pediatric populations [11, 15]. However, recent meta-analyses propose alternate cut-offs, suggesting that a single WHtR cut-off may not be universally optimal, and necessitating specific cut-offs be used in different populations [16–18].

This study aims to determine the optimal WHtR cut-offs for predicting the presence of cardiometabolic risk factors in children and adolescents from ten different populations around the world.

## Methods

### Study population

Data were from seven population-based cross-sectional surveys including eighteen public high schools in Northeastern Brazil (2012–2013) [19], the Huantai Children Cardiovascular Health Cohort (HCCHC) from one primary school in China (2017–2018) [20], a survey in five schools in the Karlovassi province of Greece (2008–2010) [21], a survey of eight primary schools in Calabria, Italy (2007–2008) [22], a school-based survey in South Africa (2007–2008) [23], a survey in primary care centers in Catalonia in Spain (2007–2014) [24], the Child Heart and Health Study in England (2006–2007) [25]; as well as three nationally representative surveys including the “Childhood and Adolescence Surveillance and Prevention of Adult Non-communicable Diseases” in Iran (2011–2012) [26], the Korean National Health and Nutrition Examination Surveys (1998–2013) [27], and the US National Health and Nutrition Examination Surveys (NHANES, 1999–2012) [28]. Table 1 summarizes basic characteristics of these surveys in ten countries.

**Table 1:** Description of surveys assessing WHtR and cardiometabolic risk factors in children and adolescents aged 6–18 years from ten countries

Country	Region	Survey year	Description	Total No	No. of boys	No. of girls	Age range (years)	Variables	WC measurement method
Brazil	South America	2012–2013	Eighteen public high schools in Campina Grande	441	141	300	15–17	Age, sex, height, weight, WC, TG, HDL, FBG, SBP, DBP	WHO recommendation
China	Asia	2017–2018	One primary school in Huantai county of Zibo city	1416	750	666	6–11	Age, sex, height, weight, WC, TG, HDL, FBG, SBP, DBP	At 1 cm above the umbilicus from the horizontal level
Greece	Europe	2008–2010	Five schools of the Karlovassi province	439	207	232	8–17	Age, sex, height, weight, WC, TG, HDL, FBG, SBP, DBP	WHO recommendation
Iran	Asia	2011–2012	National survey “Childhood and Adolescence Surveillance and Prevention of Adult Non-communicable Diseases”	8171	4047	4124	6–18	Age, sex, height, weight, WC, TG, HDL, FBG, SBP, DBP	WHO recommendation
Italy	Europe	2007–2008	Eight primary schools of Reggio Calabria	570	284	286	10–13	Age, sex, height, weight, WC, TG, HDL, FBG, SBP, DBP	The narrowest point between the lower rib and the iliac crest
Korea	Asia	2001–2013	Pooled data from 5 cycles of the Korea NHANES	7769	4111	3658	6–18	Age, sex, height, weight, WC, TG, HDL, FBG, SBP, DBP	WHO recommendation
South Africa	Africa	2007–2008	The school-based study involving fourteen schools	1271	496	775	10–16	Age, sex, height, weight, WC, TG, HDL, FBG, SBP, DBP	The narrowest part of the torso
Spain	Europe	2007–2014	Caucasian healthy children in a primary care setting in Girona	631	337	294	6–13	Age, sex, height, weight, WC, TG, HDL, FBG, SBP, DBP	The umbilical level
UK	Europe	2006–2007	Child Heart and Health Study in England (CHASE Study)	783	363	420	10–11	Age, sex, height, weight, WC, TG, HDL, FBG, SBP, DBP	WHO recommendation
USA	North America	2001–2014	NHANES	3114	1591	1523	12–17	Age, sex, height, weight, WC, TG, HDL, FBG, SBP, DBP	The high point of the iliac crest

*Abbreviations*: *DBP* Diastolic blood pressure, *FBG* Fasting blood glucose, *HDL* High-density lipoprotein, *NHANES* National Health and Nutrition Examination Survey, *SBP* Systolic blood pressure, *TG* Triglycerides, *UK* United Kingdom, *USA* United States of America, *WC* Waist circumference, *WHO* World Health Organization, *WHtR* Waist-to-height ratio

**Fig. 1:**
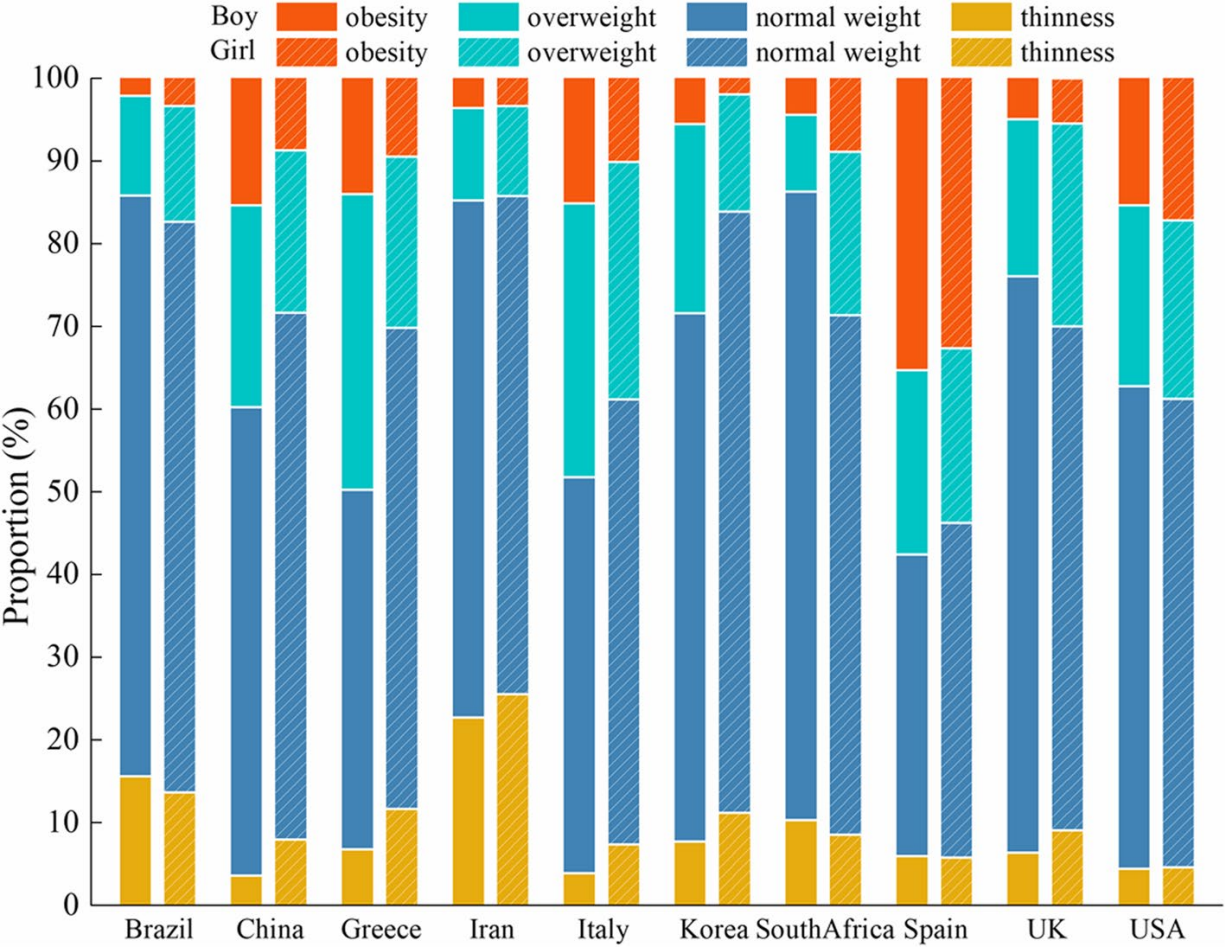
Distribution of thinness, normal weight, overweight and obesity according to IOTF BMI criteria in children and adolescents in ten countries

The weight status was assessed using the IOTF sex-and age- specific BMI percentile values [5, 29]. A wide variation in the proportion of children and adolescents classified as thin, overweight, and obese, across the ten included countries was observed (Fig. 1 and Additional file 1: Table S1). All participating surveys were granted ethical clearance by their respective institutional review boards, and informed consent was obtained from both the study participants and their parents or guardians.

### Design outline of this study

We performed two independent analyses to establish the optimal WHtR cut-offs. The first analysis strategy selected a subsample population with lower cardiometabolic risk from each country’s survey to calculate the percentile values of WHtR by age, sex, and country. Due to potential distortions caused by extreme weight conditions (high or low) in establishing weight-related reference values [4, 8, 30, 31], we used six subsamples, each incorporating different weight statuses in each country’s survey: Subsample 1 incorporated the entire population; Subsample 2, individuals with normal weight; Subsample 3, normal weight plus thinness grade 1 and overweight; Subsample 4, normal weight plus overweight and obesity; Subsample 5, normal weight plus overweight; and Subsample 6, normal weight plus overweight, obesity, and morbid obesity. Next, we sought to identify the relatively optimal percentile cut-offs of WHtR in each country’s survey using receiver operator characteristic (ROC) curve analyses to discriminate between children and adolescents who do or do not have at least two cardiometabolic risk factors. Finally, we evaluated the characteristics of these relatively optimal percentile values based on age, sex, and country, simplifying these to single static WHtR cut-offs independent of age, sex, and country.

**Fig. 2:**
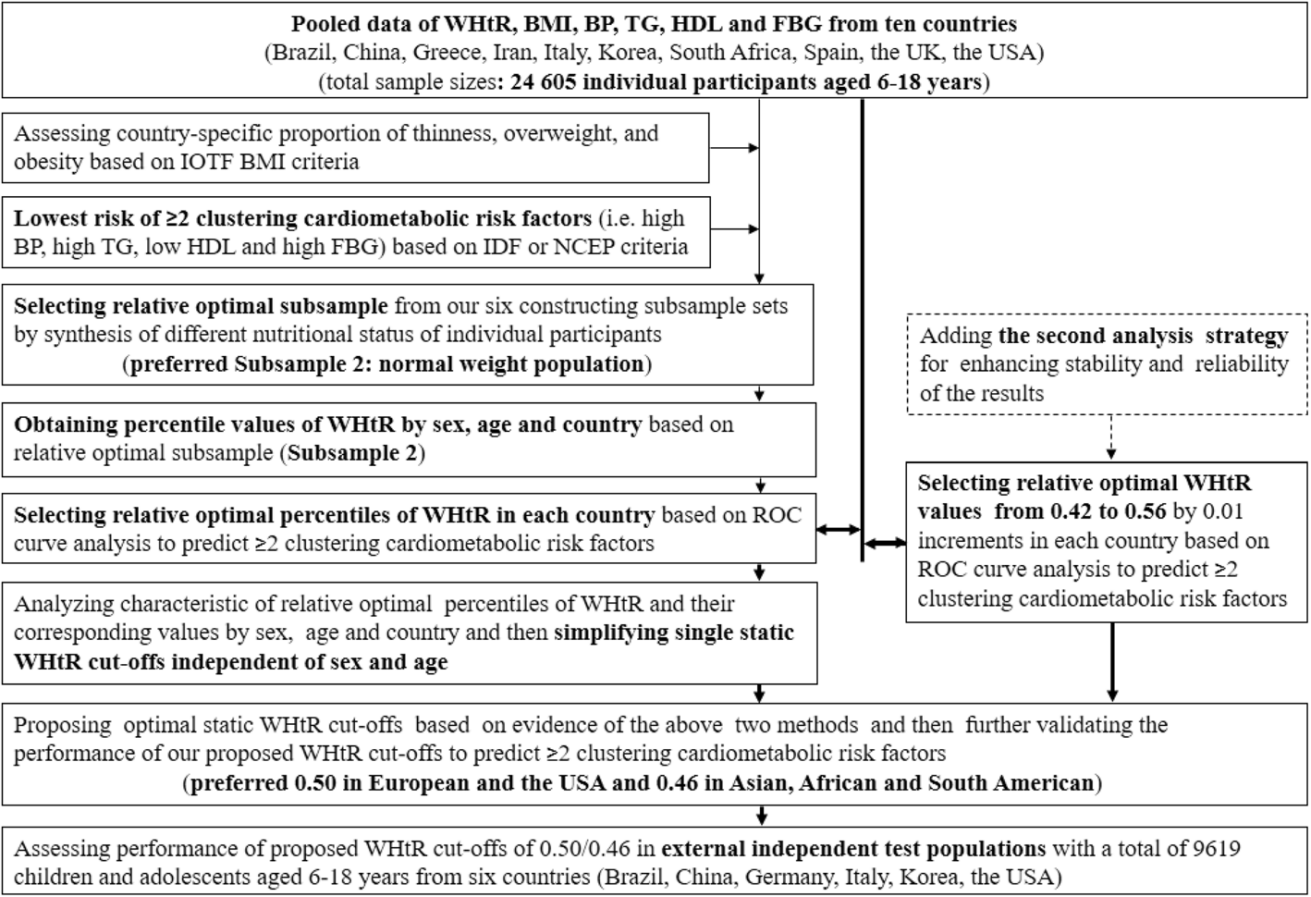
Flow chart of study design and pooled analysis

The second analysis strategy involved directly examining WHtR values ranging from 0.42 to 0.56 (with 0.01 increments) using ROC curve analyses to discriminate between those with and without the presence of two or more cardiometabolic risk factors across the ten countries. By integrating results from the two analysis strategies, we proposed relatively optimal static WHtR cut-offs, which we further validated for their utility to discriminate the presence of two or more cardiometabolic risk factors. The first analysis explored the variance of WHtR cut-offs with sex and age, aiming to determine the feasibility of simplifying these to a single cut-off. The second analysis, while more straightforward, hinged on the results from the first analysis, making the first analysis a prerequisite for the second analysis. The utility of potential cut-offs from the first and second analyses was then evaluated separately in the total sample of 24,605 participants. Finally, we further assessed the performance of proposed cut-offs in predicting at least two or at least three cardiometabolic risk factors in external independent test populations from six countries. Figure 2 shows a flow chart incorporating the study design and pooled analysis.

### Measurements

Measurements were weight, height, WC, systolic BP (SBP), diastolic BP (DBP), TG, HDL-C and FBG across the ten countries. Weight and height were measured for each child in light clothing without hat and shoes. BMI was determined by dividing weight (kilograms) by the square of height (meters). WC was measured using a non-elastic tape at the midway point between the lowest rib margin and the iliac crest in a horizontal plane at the end of a normal expiration in Brazil, Greece, Iran, Korea, and the UK as recommended by the WHO [32]; at 1 cm above the umbilicus from the horizontal level in China; at the narrowest point between the lower rib and the iliac crest in Italy; at the level of the narrowest part of the torso in South Africa; at the umbilical level in Spain; and at the high point of the iliac crest in the USA. WHtR was calculated as WC (cm) divided by height (cm).

BP was measured using auscultatory mercury sphygmomanometers in Iran, Italy, Korea and the USA; using an Omron-hem 742 semi-automatic device in Brazil; using an Omron-hem 7012 semi-automatic device in China; using an Omron 705IT semi-automated device in Greece; using a Rossmax PA semi-automatic device in South Africa; using a Dinamap Pro 100 electronic sphygmomanometer in Spain; and using an Omron 907 semi-automatic device in the UK. In all countries, SBP and DBP was measured by trained examiners according to a standardized protocol, taken from the right arm using an appropriate-sized cuff. Participants were asked to sit quietly for at least 5 min prior to measurement. Each individual had up to three BP measurements taken, with the average used.

After a 12-h overnight fast, blood samples were acquired from the antecubital vein of the study participants in Brazil, China, Greece, Iran, Italy, Korea, Spain, the UK, and the USA, and from finger-prick capillary blood in South Africa. TG, HDL-C, and FBG were measured using an auto chemistry analyzer (Hitachi Co., Tokyo, Japan) in China, Iran and Korea; using a Hitachi 911 automatic analyzer (Roche, Basel, Switzerland) in Brazil; using an autoanalyzer Menarini BT3000 Plus (Biotechnica, Italy) in Greece; using a Vitros 950 automatic analyzer (OrthoClinical Diagnostics, Raritan, USA) in Italy; using enzymatic method in the USA; using the Accutrend GCT glucometer and CardioCheckTM PA analyzer (Polymer Technology Systems, Inc. USA) in South Africa; using the hexokinase method for FBG and an automatic analyzer (ARCHITECT, Abbott Laboratories, Abbott Park, USA) for TG and HDL-C in Spain, and using the hexokinase method for FBG and an Olympus autoanalyzer for TG and HDL-C in the UK.

### Definition of cardiometabolic risk factors clustering

We defined cardiometabolic risk as the presence of two or more of the four component risk factors (high BP, high TG, low HDL-C, and high FBG) of MetS. We excluded the WC component of MetS owing to the high correlation with WHtR. For pediatric populations, MetS is often defined using either the International Diabetes Federation (IDF) criteria [33] or the modified National Cholesterol Education Program (NCEP) criteria [34]. As the two criteria use different cut-offs to define the component risk factors, we performed separate analysis for the IDF criteria and NCEP criteria. For the IDF criteria, high TG was defined as TG ≥ 150 mg/dL, low HDL-C as HDL-C < 40 mg/dL for children aged 6–15 years and < 40 mg/dL for boys and < 50 mg/dL for girls aged 16 years or older, high BP as SBP/DBP ≥ 120/80 mmHg for those aged 6–9 years [35, 36] and ≥ 130/85 mmHg for those aged 10 years or older, and high FBG as FBG ≥ 100 mg/dL. For the NCEP criteria, high TG was defined as TG ≥ 110 mg/dL, low HDL-C as HDL-C ≤ 40 mg/dL, high BP as SBP/DBP ≥ 90^th^ percentile (age-, sex-, and height-specific) using the international child BP reference [37] for those aged 6–17-years or ≥ 130/85 mmHg for those aged 18-years, and high FBG as FBG ≥ 110mg/dL.

### External independent test populations

The external independent test populations including a total of 9,619 children and adolescents aged 6–18 years came from six countries: a community project (Estação Conhecimento) in Vitória, Brazil (2014–2016) [38], the China Health and Nutrition Survey (CHNS, 2009) [39], the community-based Praeventions-Erziehungs-Programm (PEP) Family Heart Study in Germany (2000–2007) [40], a pediatric sample enrolled at the outpatient clinics of the Department of Pediatrics, Sapienza University of Rome, Italy [41], and a school-based study in Seoul, Korea (2011–2012) [42], and the NHANES (2015–2018). Details of these studies have been described elsewhere [38–42]. Each study received ethical approval from respective institutional review boards and informed consent from the study participants and their parents/guardians. Individual data on weight, height, WC, SBP, DBP, TG, HDL-C, and FBG from these six countries contributed to this present study. The same definitions on thinness, overweight and obesity were used as the IOTF criteria and on high BP, high TG, low HDL-C, and high FBG were used as the IDF or NCEP criteria. Data on anthropometric and demographic characteristics and cardiometabolic risk factors in external independent test populations from six countries are showed in Additional file 1: Table S2. In external independent test populations, we examined the predicting performance of proposed WHtR cut-offs for ≥ 2 or ≥ 3 cardiometabolic risk factors.

### Statistical analysis

Continuous data are shown as mean (standard deviation) and categorical data as n (%). First, cardiometabolic risk factor clustering was assessed among different subsamples with different nutritional status by country. Second, WHtR percentile values were calculated based on the normal weight subsample at each country by age and sex. Third, ROC curve analysis was performed, with area under the curve (AUC), sensitivity, and specificity estimated to assess the utility of WHtR percentile values (as the first analysis strategy) and static WHtR values (as the second analysis strategy) to discriminate the presence of any combination of at least two out of the four cardiometabolic risk factors according to the IDF or NCEP criteria. The AUC can range from 0 to 1. An AUC value of 0.5 would indicate the WHtR value used has no discrimination (no better than chance alone at distinguishing between those with, versus those without, two or more cardiometabolic risk factors, e.g., tossing a coin), and a value of 1 indicates perfect discrimination. The relatively optimal cut-offs of WHtR were considered based on the maximum AUC value and the Youden index (sensitivity + specificity-1). Lastly, we compared and selected those relatively optimal percentile cut-offs and static cut-offs of WHtR from the two independent analyses to ultimately propose static cut-offs and test further the utility of the proposed cut-offs to discriminate those with ≥ 2 cardiometabolic risk factors. In addition, we also performed a sensitivity analysis assessing the utility of static WHtR values from 0.42 to 0.56 (with 0.01 increments) to discriminate the presence of any combination of at least three out of the four cardiometabolic risk factors. To further assess the predicting performance of proposed cut-offs, ROC curve analysis and odds ratios (ORs) with 95% confidence intervals (CIs) were performed using the independent variable (WHtR) of proposed cut-offs and the dependent variable (cardiometabolic risk factors clustering) based on the IDF or NCEP criteria in external independent test populations from six countries. Basic data analyses and logistic regression analysis were undertaken using SAS 9.4 (SAS Institute, Cary, NC). ROC curve analyses were performed using reportROC 3.6 package running under R 4.2.2.

## Results

In the total sample population, the proportion of thinness was 13.3%, overweight was 17.1%, and obesity was 7.1%, but these proportions varied substantially across the ten countries (Fig. 1 and Additional file 1: Table S1). The proportion with ≥ 2 cardiometabolic risk factors was 7.8% based on IDF criteria, and 14.8% based on NCEP criteria, with a large variation in the proportion with high BP, high TG, low HDL-C, high FBG and their clustering across the ten countries (Additional file 1:Table S3).

**Fig. 3:**
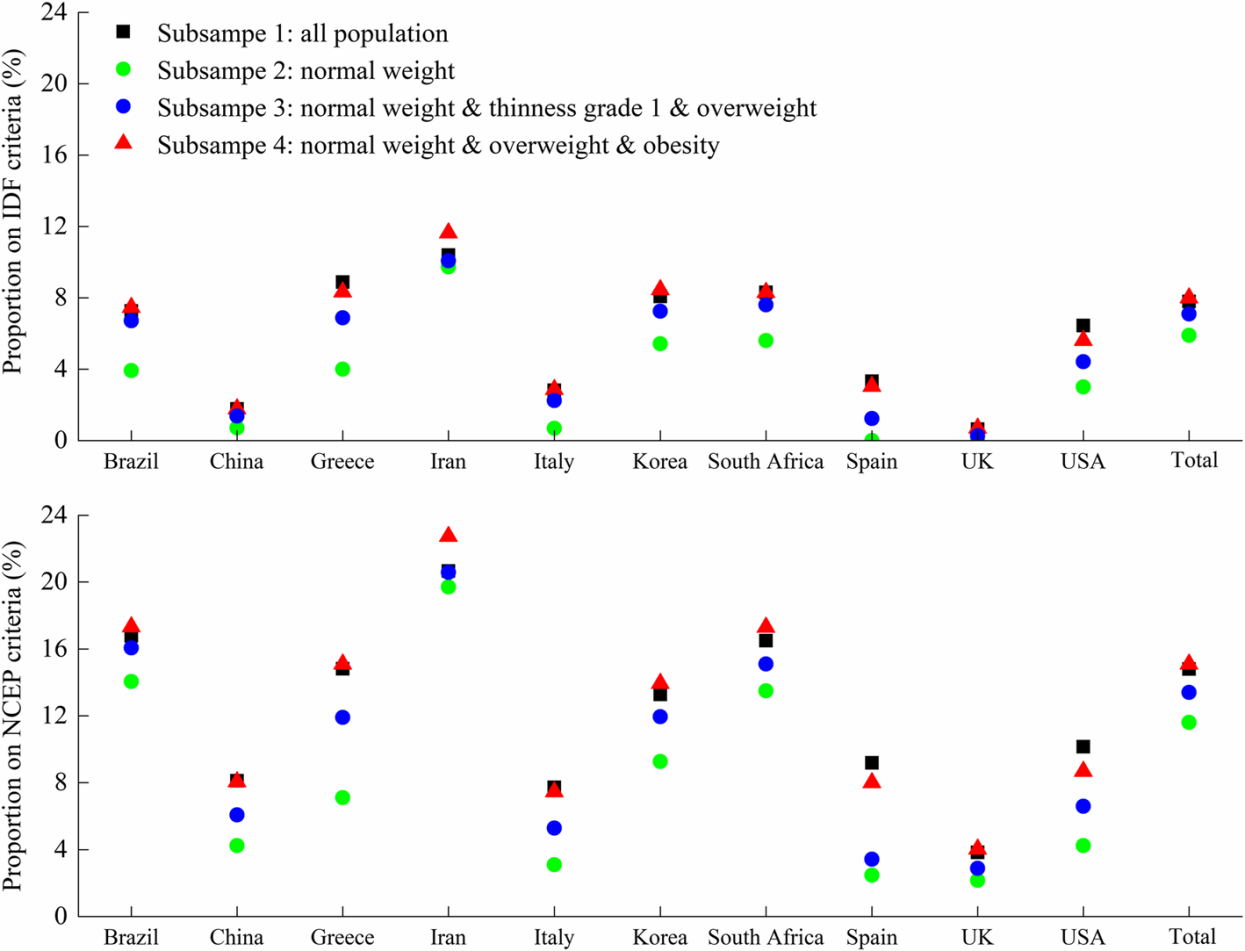
Comparison of proportion with ≥ 2 cardiometabolic risk factors among different subsamples across the ten countries

The normal weight subsample population (i.e., Subsample 2) had the lowest proportion with ≥ 2 cardiometabolic risk factors compared with the other subsample populations (thinness, overweight, obesity), according to either IDF or NCEP criteria. This was consistent across the total sample and by country (Fig. 3 and Additional file 1: Table S4). Thus, we designated the normal weight subsample as the reference population to calculate WHtR percentile reference values, based on age, sex, and country. These values were subsequently tested in the total sample of 24,605 participants.

**Fig. 4:**
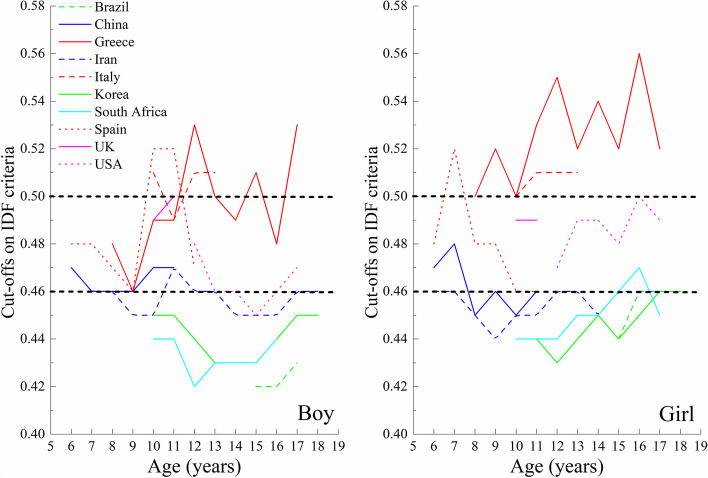
Relatively optimal percentile cut-offs of WHtR to discriminate those with and without ≥ 2 cardiometabolic risk factors using the IDF criteria for the first analysis strategy

**Fig. 5:**
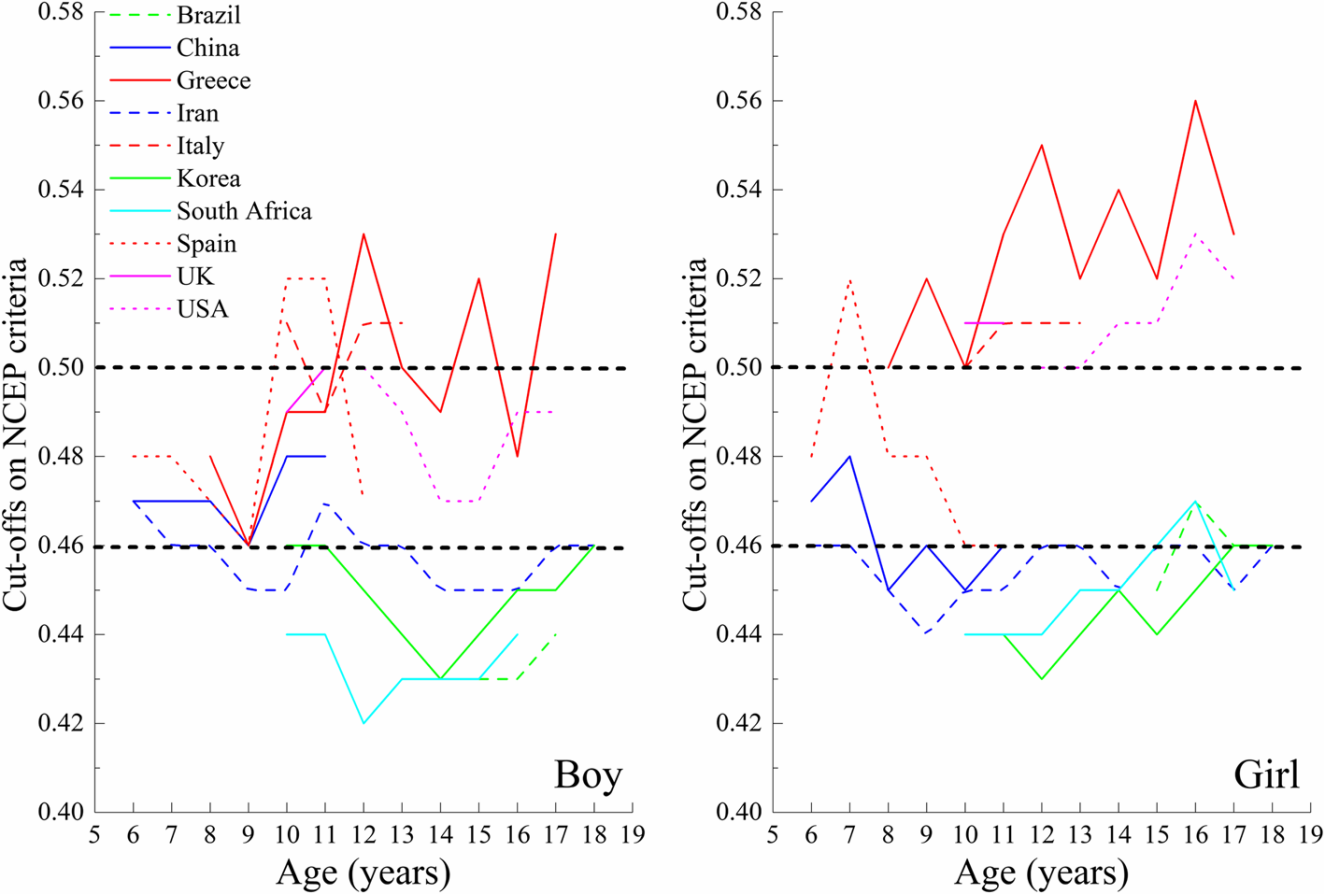
Relatively optimal percentile cut-offs of WHtR to discriminate those with and without ≥ 2 cardiometabolic risk factors using the NCEP criteria for the first analysis strategy

Based on the performance of WHtR for predicting ≥ 2 cardiometabolic risk factors using ROC curve analyses, we obtained the relatively optimal WHtR percentiles for each country (Table 2). The relatively optimal WHtR percentiles varied from P_75_ to P_95_ across the ten countries. However, the WHtR values exhibited a visible tendency to cluster irrespective of sex and the criteria used to define MetS (Figs. 4 and 5). Specifically, the value in Greece, Italy, Spain, the UK, and the USA appeared around 0.50 but a lower value (about 0.46) was seen for Brazil, China, Iran, Korea, and South Africa. In addition, these cut-offs seemed to be largely independent of age, sex, the criteria used to define MetS components, and the position of the WC measurement.

**Table 2:** Results of ROC curve analyses to identify relatively optimal percentile cut-offs of WHtR based on age-, sex-, and country-specific percentiles to discriminate those with ≥ 2 cardiometabolic risk factors, stratified by country

Country	Age (years)	Sex	No. of participants	IDF criteria				NCEP criteria			
				Cut-offs	AUC (95% CI)	Sensitivity	Specificity	Cut-offs	AUC (95% CI)	Sensitivity	Specificity
Brazil	15–17	Boys	141	P_75_	0.830 (0.715–0.946)	0.917	0.744	P_80_	0.631 (0.516–0.745)	0.465	0.796
		Girls	300	P_80_	0.654 (0.520–0.788)	0.600	0.707	P_85_	0.643 (0.530–0.756)	0.516	0.770
China	6–11	Boys	750	P_85_	0.725 (0.622–0.827)	0.909	0.540	P_90_	0.663 (0.588–0.739)	0.724	0.603
		Girls	666	P_90_	0.778 (0.609–0.888)	0.857	0.699	P_90_	0.655 (0.574–0.737)	0.596	0.714
Greece	8–17	Boys	207	P_90_	0.609 (0.484–0.734)	0.643	0.575	P_95_	0.633 (0.523–0.744)	0.650	0.617
		Girls	232	P_90_	0.723 (0.562–0.885)	0.727	0.719	P_90_	0.712 (0.591–0.833)	0.680	0.744
Iran	6–18	Boys	4047	P_75_	0.582 (0.550–0.615)	0.449	0.716	P_75_	0.561 (0.537–0.585)	0.395	0.727
		Girls	4124	P_75_	0.592 (0.562–0.621)	0.455	0.729	P_75_	0.588 (0.564–0.613)	0.434	0.743
Italy	10–13	Boys	284	P_90_	0.635 (0.474–0.796)	0.727	0.542	P_90_	0.716 (0.621–0.811)	0.857	0.574
		Girls	286	P_85_	0.792 (0.763–0.821)	1	0.584	P_85_	0.605 (0.457–0.753)	0.625	0.585
Korea	6–18	Boys	4111	P_80_	0.693 (0.662–0.723)	0.753	0.632	P_85_	0.680 (0.654–0.706)	0.666	0.694
		Girls	3658	P_80_	0.656 (0.619–0.693)	0.585	0.728	P_80_	0.538 (0.605–0.670)	0.542	0.733`
South Africa	10–16	Boys	496	P_80_	0.682 (0.601–0.763)	0.597	0.767	P_80_	0.621 (0.552–0.690)	0.471	0.772
		Girls	775	P_90_	0.632 (0.541–0.723)	0.558	0.706	P_90_	0.606 (0.542–0.670)	0.491	0.721
Spain	6–13	Boys	337	P_95_	0.695 (0.590–0.801)	0.917	0.474	P_95_	0.730 (0.666–0.793)	0.963	0.497
		Girls	294	P_95_	0.749 (0.720–0.778)	1	0.498	P_95_	0.680 (0.585–0.775)	0.839	0.521
UK	10–11	Boys	363	P_95_	0.887 (0.865–0.908)	1	0.773	P_95_	0.597 (0.436–0.758)	0.417	0.778
		Girls	420	P_90_	0.853 (0.831–0.875)	1	0.707	P_95_	0.710 (0.578–0.842)	0.611	0.808
USA	12–17	Boys	1591	P_85_	0.710 (0.664–0.756)	0.811	0.610	P_95_	0.722 (0.679–0.764)	0.711	0.733
		Girls	1523	P_80_	0.671 (0.611–0.731)	0.797	0.545	P_95_	0.704 (0.649–0.760)	0.714	0.695

*Abbreviations AUC* Area under the curve, *CI* Confidence interval, *IDF* International Diabetes Federation, *NCEP* National Cholesterol Education Program, *P* Percentile, *ROC* Receiver operator characteristic, *UK* United Kingdom, *USA* United States of America, *WHtR* Waist-to-height ratio

**Fig. 6:**
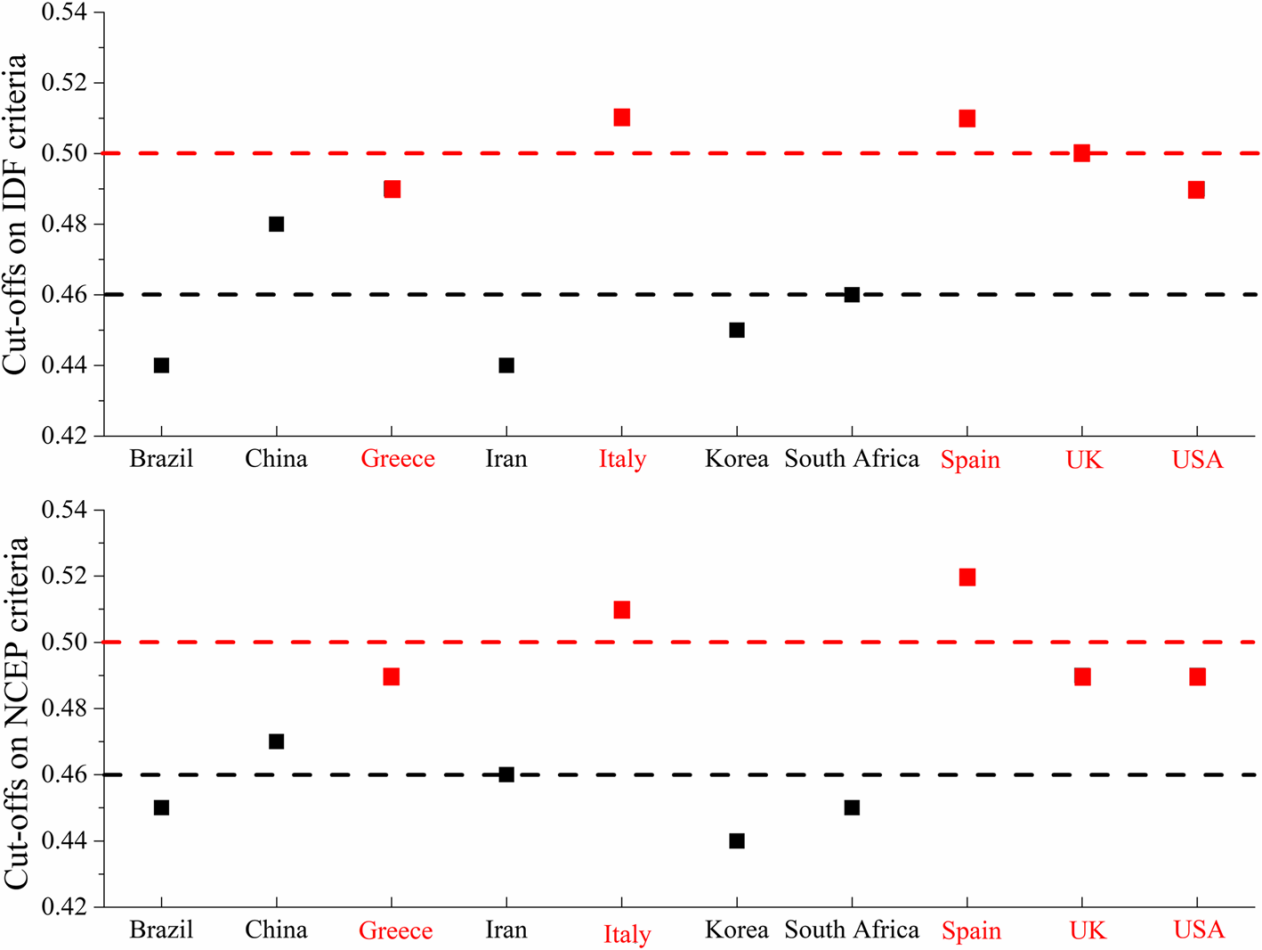
Relatively optimal cut-offs of WHtR to discriminate those with and without ≥ 2 cardiometabolic risk factors for the second analysis strategy

In agreement with the first analysis strategy, the clustering tendency of the relatively optimal WHtR cut-offs was also observed in the second analysis strategy, with values around 0.50 for Greece, Italy, Spain, the UK, and the USA, and around 0.46 for Brazil, China, Iran, Korea, and South Africa (Fig. 6 and Additional file 1: Table S5).

We tested the performance of the cut-offs of 0.50 and 0.46 to discriminate children and adolescents with and without ≥ 2 cardiometabolic risk factors across the ten countries (Table 3). Irrespective of the criteria used to define the MetS components and the measurement position for WC, we found that a WHtR cut-off of 0.50 is optimal in populations from Europe and the USA, and a cut-off of 0.46 is optimal in populations from Asia, Africa, and South America (Table 3). In addition, in sensitivity analysis for discriminating between those with and those without ≥ 3 cardiometabolic risk factors, we found that a WHtR cut-off of 0.46 is optimal in populations from Asia, Africa, and South America, according to either IDF or NCEP criteria, and a cut-off of 0.50 is optimal in populations from Europe and the USA, according to IDF criteria, but a cut-off of 0.52 is optimal in populations from Europe and the USA, according to NCEP criteria (Additional file 1: Table S6).

**Table 3:** Results of ROC curve analyses that test the proposed relatively optimal WHtR cut-offs of 0.50 and 0.46 to discriminate those with ≥ 2 cardiometabolic risk factors, stratified by country

Country	Age (years)	No. of subjects	Cut-offs	IDF criteria			NCEP criteria		
				AUC (95% CI)	Sensitivity	Specificity	AUC (95% CI)	Sensitivity	Specificity
Brazil	15–17	441	0.46	0.680 (0.574–0.785)	0.562	0.797	0.565 (0.491–0.640)	0.338	0.793
			0.50	0.626 (0.534–0.718)	0.312	0.939	0.574 (0.517–0.632)	0.203	0.946
China	6–11	1416	0.46	0.731 (0.655–0.808)	0.880	0.582	0.652 (0.597–0.707)	0.704	0.599
			0.50	0.702 (0.597–0.807)	0.640	0.764	0.661 (0.604–0.718)	0.539	0.783
Greece	8–17	439	0.46	0.624 (0.559–0.689)	0.923	0.325	0.624 (0.564–0.683)	0.908	0.340
			0.50	0.626 (0.531–0.722)	0.718	0.535	0.647 (0.569–0.726)	0.738	0.566
Iran	6–18	8171	0.46	0.587 (0.565–0.609)	0.425	0.749	0.578 (0.561–0.595)	0.393	0.763
			0.50	0.578 (0.560–0.597)	0.261	0.895	0.565 (0.552–0.579)	0.225	0.906
Italy	10–13	570	0.46	0.605 (0.527–0.683)	0.938	0.273	0.595 (0.534–0.657)	0.909	0.281
			0.50	0.663 (0.547–0.780)	0.812	0.514	0.651 (0.567–0.734)	0.773	0.529
Korea	6–18	7769	0.46	0.668 (0.644–0.693)	0.572	0.765	0.653 (0.633–0.673)	0.527	0.778
			0.50	0.607 (0.585–0.629)	0.317	0.897	0.612 (0.594–0.629)	0.314	0.910
South Africa	10–16	1271	0.46	0.635 (0.575–0.695)	0.514	0.756	0.608 (0.562–0.655)	0.448	0.769
			0.50	0.588 (0.534–0.642)	0.305	0.871	0.583 (0.542–0.623)	0.281	0.884
Spain	6–13	631	0.46	0.684 (0.618–0.749)	0.952	0.487	0.665 (0.605–0.724)	0.897	0.433
			0.50	0.730 (0.648–0.813)	0.905	0.556	0.731 (0.669–0.793)	0.879	0.583
UK	10–11	783	0.46	0.801 (0.784–0.818)	1	0.602	0.655 (0.555–0.754)	0.710	0.610
			0.50	0.894 (0.880–0.908)	1	0.788	0.664 (0.561–0.768)	0.553	0.795
USA	12–17	3114	0.46	0.658 (0.623–0.694)	0.826	0.491	0.665 (0.635–0.695)	0.826	0.504
			0.50	0.670 (0.629–0.711)	0.662	0.678	0.691 (0.657–0.725)	0.687	0.695

*Abbreviations AUC* Area under the curve, *CI* Confidence interval, *IDF* International Diabetes Federation, *NCEP* National Cholesterol Education Program, *P* Percentile, *ROC* Receiver operator characteristic, *UK* United Kingdom, *USA* United States of America, *WHtR* Waist-to-height ratio

We further tested the performance of the proposed cut-offs 0.50 and 0.46 for two different pediatric populations, respectively, in external independent test populations from six countries, showing a remarkable ability in predicting ≥ 2 cardiometabolic risk factors clustering, with ORs (95% CIs) of 3.49 (0.99–12.30) in Brazil, 4.02 (2.15–7.51) in China, 3.20 (2.47–4.14) in Germany, 5.19 (2.06–13.08) in Italy, 5.09 (2.05–12.64) in Korea and 3.34 (1.74–6.42) in the USA based on the IDF criteria, and with ORs (95% CIs) of 3.23 (1.89–5.55) in Brazil, 2.12 (1.30–2.46) in China, 2.99 (2.39–3.74) in Germany, 3.46 (2.08–5.76) in Italy, 4.78 (1.86–12.28) in Korea and 3.28 (1.60–6.74) in the USA based on NCEP criteria (Table 4). Similar results were observed when predicting ≥ 3 cardiometabolic risk factors clustering (Table 4). In addition, ROC curve analysis based on external independent six test populations also confirmed similar results with the proposed cut-offs 0.50 and 0.46 for two different pediatric populations (Additional file 1: Table S7).

**Table 4:** Performance of proposed WHtR cut-offs of 0.50/0.46 in external independent test pediatric populations aged 6–18 years from six countries

	Brazil (0.46)	China (0.46)	Germany (0.50)	Italy (0.50)	Korea (0.46)	USA (0.50)	Total
Survey year	2014–2016	2009	2000–2007	2008–2010	2011–2012	2015–2018	2000–2018
Sample size	824	749	6810	724	152	360	9619
Boys (%)	463 (56.2)	411 (54.9)	3578 (52.5)	395 (54.6)	105 (69.1)	179 (49.7)	5131 (53.3)
Age (years)	6–18	7–17	6–18	6–18	12–15	12–18	6–18
Cardiometabolic risk factors clustering based on IDF criteria, OR (95% CI)							
≥ 2	3.49 (0.99–12.30)	4.02 (2.15–7.51)	3.20 (2.47–4.14)	5.19 (2.06–13.08)	5.09 (2.05–12.64)	3.34 (1.74–6.42)	3.70 (3.09–4.43)
≥ 3	3.39 (0.21–54.52)	2.33 (0.39–14.07)	4.04 (1.97–8.29)	5.42 (0.71–41.52)	2.62 (0.31–22.52)	7.83 (0.90–67.81)	5.40 (3.34–8.74)
Cardiometabolic risk factors clustering based on NCEP criteria, OR (95% CI)							
≥ 2	3.23 (1.89–5.55)	2.12 (1.30–2.46)	2.99 (2.39–3.74)	3.46 (2.08–5.76)	4.78 (1.86–12.28)	3.28 (1.60–6.74)	4.03 (3.48–4.66)
≥ 3	7.16 (1.77–28.94)	2.08 (0.49–8.80)	6.18 (3.86–9.90)	9.13 (2.19–38.15)	5.73 (0.70–46.64)	6.06 (0.67–54.86)	8.23 (5.87–11.55)

*Abbreviations CI* Confidence interval, *IDF* International Diabetes Federation, *NCEP* National Cholesterol Education Program, *OR* Odds ratio, *WHtR* Waist-to-height ratio. OR (95%CI) was calculated using < 2 cardiometabolic risk factors clustering as the reference group

Mutivariate logistic regression models were adjusted for sex and age in each population, and additionally adjusted for survey year in total population

## Discussion

This is, to our knowledge, the first study to thoroughly investigate the universality of the message, “keep your waist less than half of your height”, amongst children and adolescents in a large, multinational sample involving ten countries from five WHO regions. We found that a WHtR cut-off of 0.50 might be suitable for evaluating cardiometabolic risk in children and adolescents from Europe and the USA, while a lower value of 0.46 might be more appropriate for those from Asia, Africa, and South America. These cut-offs were found to be largely independent of age, sex, the specific criteria used to define MetS, and the WC measurement position.

We observe that the mean levels of WHtR are stable in children and adolescents aged 6–18 years across the different samples studied. Despite there are variations in the WHtR distributions across the samples studied, the variations appear non-significant. Our primary objective was thus to establish a simple and universally applicable WHtR value for identifying children and adolescents at heightened cardiometabolic risk, as well as to make comparisons of this WHtR value’s performance across different countries. Despite the diversity in our samples, we found visual evidence for the best WHtR cut-offs tending to cluster across the ten countries. Although higher cut-offs (such as 0.55) have been suggested to screen children at higher cardiometabolic risk [43], our data argue against the universal application of these higher thresholds for predicting cardiometabolic risk in pediatric populations. Furthermore, while a WHtR cut-off of 0.50 is commonly used, our findings underscore that a single cut-off value may not be optimal if applied to all countries. Our proposed WHtR cut-off of 0.50 for European and US children and adolescents aligns with the KiGGS Study in Germany [44], a sample of Mexican children [45], a prospective birth cohort in Australia [46], and the cut-off that is most commonly used [12]. However, our proposed cut-off of 0.46 for Asian, African, and South American children and adolescents resonates with a recent meta-analysis which suggested an appropriate cut-off of 0.46 for children and adolescents from East and Southeast Asia [18].

Although determination of the most appropriate WHtR cut-offs poses numerous challenges, we strived to find and select such cut-offs based on the best (feasible and technical) evidence-based approach. First, abnormal percentile distribution of weight-related indicators such as BMI, WC, WHtR often occur due to adverse nutritional statuses in a population, particularly the high proportion of overweight and obesity. To circumvent the issue of “unhealthy” weight, akin to the approach taken in constructing the WHO growth standard/reference [4, 47], international WC percentile reference [8], and European body composition percentile reference from the IDEFICS study [30], we selected a relatively optimal subsample from our six constructed subsample sets, each corresponding to participants with different nutritional statuses in each country. Ultimately, we adopted the normal weight subsample to calculate WHtR percentile values due to it having the lowest proportion of cardiometabolic risk factors clustering. These WHtR percentile values were then employed in ROC curve analyses to determine the relatively optimal WHtR cut-offs to discern those with two or more cardiometabolic risk factors in our first analysis strategy. However, we acknowledge that this method of data processing, while common, is also a compromise in the face of the high levels of overweight and obesity in contemporary children and adolescents. To enhance the robustness and reliability of our primary analysis strategy, we considered a secondary analysis strategy that directly screened relatively optimal WHtR values within the range of 0.42 to 0.56, incrementing by 0.01.

Second, although the WHO recommends WC measurements be made on a horizontal plane at the midway point between the lowest rib margin and the iliac crest, protocol variations exist. For example, data from several relatively small studies suggest WC measurements that are 3–4 cm larger when taken above the iliac crest compared to those taken at the midway or narrowest point between the lowest rib and the iliac crest [48, 49]. Although WC measurement position tends to have reduced impact on WHtR (about 0.02), we took care to avoid mixing data from each country in the analysis stage to limit heterogeneity.

Third, the close association of WHtR with each component of the MetS, albeit weaker with BP [50, 51], is worth consideration. For example, a recent study from Spain among 8–11 year old showed that different WHtR cut-offs depended on the outcome measure (≥ 0.57 for high BP, ≥ 0.52 for high TG, ≥ 0.51 for MetS) [52]. Although different cardiometabolic risk factors might correlate with specific WHtR cut-offs, we concentrated our efforts on exploring relatively optimal WHtR cut-offs to identify those with cardiometabolic risk factor clustering. This approach aligns with the notion that combined consideration of high WHtR and other risk factors might provide more practical guidance for screening MetS or cardiometabolic risk in pediatric populations.

We determined relatively optimal WHtR cut-offs based on their discriminatory ability for the presence of ≥ 2 cardiometabolic risk factors using ROC curve analyses. Overall, the discriminatory ability of WHtR alone was approximately moderate (< 0.70). However, these values need to be put into context of cardiometabolic risk in population settings. For example, a systematic review showed that the mean AUC values were 0.70, 0.69, and 0.67, for WHtR, WC, and BMI, respectively, for predicting risk of diabetes and cardiovascular disease in adults [14]. Further, the Framingham Risk Score (FRS), based on traditional adult risk factors of age, sex, BMI, TC, HDL-C, SBP, smoking, and diabetes, has AUC values ranging from 0.70 to 0.85 in most populations but is used in clinical settings to predict an individual’s future risk of cardiovascular disease [53]. Although we observed low AUC values in some countries and analyses, we nonetheless observed a tendency for the optimal WHtR cut-offs to cluster across the various populations in our sample. This observation was consistent throughout the analysis strategy used or alternate definitions of cardiometabolic risk. These findings highlight that simple indicators, even those based on combined measures such as FRS, may not be good enough to accurately discriminate or predict strong outcomes when used in isolation. However, given the inherent convenience of WHtR, its use, especially in conjunction with other measures (e.g., BP, blood lipids, blood glucose, family history of cardiovascular disease), could be potentially valuable for identifying children and adolescents at elevated cardiometabolic risk, although this assumption was not specifically examined in our study. In future clinical and public health practice, WHtR could be considered for application in the definition of MetS to replace the use of WC percentile based on sex and age, as WHtR is simple and convenient which allows for easy and rapid identification of children with potential metabolic risk.

### Strengths and limitations of this study

Our study has several strengths. First, we had a large sample of participants from ten countries representing five of the six WHO regions. Second, we considered two different MetS criteria to define the cardiometabolic risk factors, with both producing consistent results. Third, our comprehensive analysis strategy that sought to enhance the robustness of our findings, yielded consistent results. Fourth, we used external independent test populations to further assess the predicting performance of proposed cut-offs for cardiometabolic risk factors clustering. Our study is not without limitations. First, since our results are based on cross-sectional data, we were unable to determine causation in the studied relationships, thus necessitating further investigation via prospective cohort studies. However, longitudinal research has suggested that adiposity may be the initial factor leading to the clustering of other cardiometabolic risk components [54–56]. Second, although the role of WC measurement methods on WHtR cut-offs needs further understanding, variation is likely in real-world settings. In this respect, it was reassuring that we observed the tendency for our WHtR cut-offs to cluster from the ten countries, irrespective of the WC measurement method used. Third, while we investigated relatively optimal WHtR cut-offs in discriminating children and adolescents with clustered cardiometabolic risk factors, further studies should validate these proposed cut-offs in relation to abdominal fat in predicting cardiometabolic risk using longitudinal data. Fourth, the age range of the included study populations varied across different countries. However, instead of arbitrarily mixing these data, we used them separately which performed robustly across the age range. Fifth, the BP measurement devices in most of the studies hadn’t been validated for use in children and adolescents according to the STRIDE BP validation, which may affect the accuracy of BP measurements. Sixth, we defined high BP using the validated static BP cut-offs recommended by the IDF, nonetheless, BP normogram for each population may also be better when each population has the corresponding national reference. However, it is a pity that most of the included countries do not have the BP normogram for each population.

## Conclusions

Our findings suggest that a WHtR cut-off of 0.50 may be appropriate to evaluate cardiometabolic risk in children and adolescents from Europe and the USA, while a lower cut-off (0.46) may be suitable for those from Asia, Africa, and South America. These cut-offs were largely independent of age, sex, MetS criteria, and WC measurement position. Therefore, they could serve as potential thresholds for dichotomizing WHtR in predicting cardiometabolic risk among children and adolescents from diverse populations.

### Supplementary Information

**Additional file 1:  Table S1.** Proportions of children and adolescents from ten countries classified according to IOTF BMI criteria. **Table S2.** Characteristics of external independent test pediatric populations aged 6-18 years from six countries. **Table S3.** Proportions of children and adolescents from ten countries with single and clustered cardiometabolic risk factors based on IDF and NCEP criteria. **Table S4.** Comparison of proportion of children and adolescents among different subsamples from ten countries with ≥2 cardiometabolic risk factors based on IDF and NCEP criteria. **Table S5.** Results from ROC curve analyses to identify relatively optimal cut-offs of WHtR to discriminate those with ≥2 cardiometabolic risk factors from ten countries using the second analysis strategy. **Table S6.** Results from ROC curve analyses to identify relatively optimal cut-offs of WHtR to discriminate those with ≥3 cardiometabolic risk factors from ten countries. **Table S7.** Results from ROC curve analyses to identify relatively optimal cut-offs of WHtR to discriminate those with ≥2 cardiometabolic risk factors in external independent test pediatric populations from six countries.

## Data Availability

The data that support the findings of this study are available from the cor‑responding author upon reasonable request.

## Abbreviations

AUC: Area under the curve
BMI: Body mass index
CI: Confidence interval
DBP: Diastolic blood pressure
FBG: Fasting blood glucose
HDL: High-density lipoprotein
IDF: International Diabetes Federation
IOTF: International Obesity Task Force
MetS: Metabolic syndrome
NCEP: National Cholesterol Education Program
NHANES: National Health and Nutrition Examination Survey
OR: Odds ratio
ROC: Receiver operator characteristic
SBP: Systolic blood pressure
TG: Triglycerides
UK: United Kingdom
USA: United States of America
WC: Waist circumference
WHO: World Health Organization
WHtR: Waist-to-height ratio
