# Supplementary material for: Establishing international optimal cut-offs of waist-to-height ratio for predicting cardiometabolic risk in children and adolescents aged 6–18 years
Source: BMC Med. 2023 Nov 15;21:442. doi: 10.1186/s12916-023-03169-y (PMC10647138; doi:10.1186/s12916-023-03169-y)
Supplement: Supplementary file 1 — Additional file 1: Table S1. Proportions of children and adolescents from ten countries classified according to IOTF BMI criteria. Table S2. Characteristics of external independent test pediatric populations aged 6-18 years from six countries. Table S3. Proportions of children and adolescents from ten countries with single and clustered cardiometabolic risk factors based on IDF and NCEP criteria. Table S4. Comparison of proportion of children and adolescents among different subsamples from ten countries with ≥2 cardiometabolic risk factors based on IDF and NCEP criteria. Table S5. Results from ROC curve analyses to identify relatively optimal cut-offs of WHtR to discriminate those with ≥2 cardiometabolic risk factors from ten countries using the second analysis strategy. Table S6. Results from ROC curve analyses to identify relatively optimal cut-offs of WHtR to discriminate those with ≥3 cardiometabolic risk factors from ten countries. Table S7. Results from ROC curve analyses to identify relatively optimal cut-offs of WHtR to discriminate those with ≥2 cardiometabolic risk factors in external independent test pediatric populations from six countries. [file 12916_2023_3169_MOESM1_ESM.doc]

**Table S1** Proportions of children and adolescents from ten countries classified according to IOTF BMI criteria

| Country | Age  (years) | Sample size | Thinness grade 3,  n (%) | Thinness grade 2,  n (%) | Thinness grade 1,  n (%) | Normal weight,  n (%) | Overweight,  n (%) | Obesity,  n (%) | Morbid obesity,  n (%) |
| --- | --- | --- | --- | --- | --- | --- | --- | --- | --- |
| Brazil | 15-17 | 441 | 0 | 11 (2.5) | 52 (11.8) | 306 (69.4) | 59 (13.4) | 10 (2.2) | 3 (0.7) |
| China | 6-11 | 1416 | 1 (0.1) | 8 (0.6) | 71 (5.0) | 849 (59.9) | 314 (22.2) | 128 (9.0) | 45 (3.2) |
| Greece | 8-17 | 439 | 3 (0.7) | 7 (1.6) | 31 (7.1) | 225 (51.2) | 122 (27.8) | 37 (8.4) | 14 (3.2) |
| Iran | 6-18 | 8171 | 257 (3.1) | 449 (5.5) | 1266 (15.5) | 5014 (61.4) | 901 (11.0) | 236 (2.9) | 48 (0.6) |
| Italy | 10-13 | 570 | 3 (0.5) | 3 (0.5) | 26 (4.6) | 290 (50.9) | 176 (30.9) | 57 (10.0) | 15 (2.6) |
| Korea | 6-18 | 7769 | 38 (0.5) | 117 (1.5) | 571 (7.3) | 5285 (68.0) | 1459 (18.8) | 268 (3.5) | 31 (0.4) |
| South Africa | 10-16 | 1271 | 4 (0.3) | 8 (0.6) | 105 (8.2) | 864 (68.0) | 199 (15.7) | 66 (5.2) | 25 (2.0) |
| Spain | 6-13 | 631 | 5 (0.8) | 3 (0.5) | 29 (4.6) | 242 (38.4) | 137 (21.7) | 146 (23.1) | 69 (10.9) |
| UK | 10-11 | 783 | 3 (0.4) | 9 (1.1) | 49 (6.3) | 509 (65.0) | 172 (22.0) | 38 (4.8) | 3 (0.4) |
| USA | 12-17 | 3114 | 9 (0.3) | 18 (0.6) | 113 (3.6) | 1792 (57.6) | 676 (21.7) | 306 (9.8) | 200 (6.4) |
| Total | 6-18 | 24605 | 323 (1.3) | 633 (2.6) | 2313 (9.4) | 15376 (62.5) | 4215 (17.1) | 1292 (5.3) | 453 (1.8) |

*Abbreviations BMI* body mass index, *IOTF* International Obesity Task Force, U*K* United Kingdom, *USA* United States of America.

**Table S2** Characteristics of external independent test pediatric populations aged 6-18 years from six countries

|  | Brazil | China | Germany | Italy | Korea | USA | Total |
| --- | --- | --- | --- | --- | --- | --- | --- |
| ***Survey year*** | 2014-2016 | 2009 | 2000-2007 | 2008-2010 | 2011-2012 | 2015-2018 | 2000-2018 |
| ***Sample size*** | 824 | 749 | 6810 | 724 | 152 | 360 | 9619 |
| Boys (%) | 463 (56.2) | 411 (54.9) | 3578 (52.5) | 395 (54.6) | 105 (69.1) | 179 (49.7) | 5131 (53.3) |
| ***Age (years)*** | 6-18 | 7-17 | 6-18 | 6-18 | 12-15 | 12-18 | 6-18 |
| ***Weight status*** |  |  |  |  |  |  |  |
| Thinness, n (%) | 81 (9.8) | 163 (21.8) | 603 (8.9) | 8 (1.1) | 0 | 17 (4.7) | 872 (9.1) |
| Normal weight, n (%) | 535 (64.9) | 494 (65.9) | 5182 (76.1) | 140 (19.3) | 0 | 184 (51.1) | 6535 (67.9) |
| Overweight, n (%) | 150 (18.2) | 75 (10.0) | 841 (12.3) | 232 (32.0) | 113 (74.3) | 80 (22.2) | 1491 (15.5) |
| Obesity, n (%) | 58 (7.1) | 17 (2.3) | 184 (2.7) | 344 (47.5) | 39 (25.7) | 79 (21.9) | 721 (7.5) |
| **High WHtR, n (%)** | 195 (23.7) | 180 (24.0) | 747 (11.0) | 537 (74.2) | 111 (73.0) | 154 (42.8) | 1924 (20.0) |
| ***IDF criteria*** |  |  |  |  |  |  |  |
| High BP, n (%) | 19 (2.3) | 35 (4.7) | 412 (6.1) | 93 (12.9) | 55 (36.2) | 13 (3.6) | 627 (6.5) |
| High TG, n (%) | 16 (1.9) | 75 (10.0) | 125 (1.8) | 74 (10.2) | 30 (19.7) | 21 (5.8) | 341 (3.6) |
| Low HDL, n (%) | 176 (21.4) | 72 (9.6) | 551 (8.1) | 143 (19.8) | 25 (16.5) | 58 (16.1) | 1025 (10.7) |
| High FBG, n (%) | 32 (3.9) | 61 (8.1) | 1807 (26.5) | 13 (1.8) | 9 (5.9) | 144 (40.0) | 2066 (21.5) |
| ≥2 cardiometabolic risk factors clustering, n (%) | 20 (2.4) | 43 (5.7) | 327 (4.8) | 72 (9.9) | 27 (17.8) | 47 (13.1) | 536 (5.6) |
| ≥3 cardiometabolic risk factors clustering, n (%) | 2 (0.2) | 5 (0.7) | 35 (0.5) | 15 (2.1) | 7 (4.6) | 6 (1.7) | 70 (0.7) |
| ***NCEP criteria*** |  |  |  |  |  |  |  |
| High BP, n (%) | 101 (12.3) | 143 (19.1) | 1903 (27.9) | 302 (41.7) | 99 (65.1) | 40 (11.1) | 2588 (26.9) |
| High TG, n (%) | 74 (9.0) | 176 (23.5) | 495 (7.3) | 183 (25.3) | 59 (38.8) | 58 (16.1) | 1045 (10.9) |
| Low HDL, n (%) | 169 (20.5) | 67 (9.0) | 471 (6.9) | 139 (19.2) | 25 (16.5) | 40 (11.1) | 911 (9.5) |
| High FBG, n (%) | 4 (0.5) | 15 (2.0) | 416 (6.1) | 4 (0.6) | 2 (1.3) | 19 (5.3) | 460 (4.8) |
| ≥2 cardiometabolic risk factors clustering, n (%) | 59 (7.2) | 79 (10.6) | 472 (6.9) | 170 (23.5) | 56 (36.8) | 38 (10.6) | 874 (9.1) |
| ≥3 cardiometabolic risk factors clustering, n (%) | 9 (1.1) | 8 (1.1) | 74 (1.1) | 44 (6.1) | 11 (7.2) | 5 (1.4) | 151 (1.6) |

*Abbreviations BP* blood pressure, *FBG* fasting blood glucose, *HDL* high-density lipoprotein, *IDF* International Diabetes Federation, *NCEP* National Cholesterol Education Program, *TG* triglycerides.

**Table S3** Proportions of children and adolescents from ten countries with single and clustered cardiometabolic risk factors based on IDF and NCEP criteria

| Country | Age  (years) | Sample size | High BP,  n (%) | High TG,  n (%) | Low HDL,  n (%) | High FBG,  n (%) | ≥2 cardiometabolic risk factors clustering,  n (%) | ≥3 cardiometabolic risk factors clustering,  n (%) |
| --- | --- | --- | --- | --- | --- | --- | --- | --- |
| ***IDF criteria*** | | | | | | | | |
| Brazil | 15-17 | 441 | 23 (5.2) | 26 (5.9) | 279 (63.3) | 0 | 32 (7.3) | 3 (0.7) |
| China | 6-11 | 1416 | 71 (5.0) | 65 (4.6) | 28 (2.0) | 100 (7.1) | 25 (1.8) | 1 (0.1) |
| Greece | 8-17 | 439 | 61 (13.9) | 20 (4.6) | 48 (10.9) | 77 (17.5) | 39 (8.9) | 5 (1.1) |
| Iran | 6-18 | 8171 | 518 (6.3) | 752 (9.2) | 3422 (41.9) | 631 (7.7) | 849 (10.4) | 95 (1.2) |
| Italy | 10-13 | 570 | 15 (2.6) | 23 (4.0) | 88 (15.4) | 8 (1.4) | 16 (2.8) | 0 |
| Korea | 10-18 | 7769 | 250 (3.2) | 757 (9.7) | 1760 (22.7) | 703 (9.1) | 628 (8.1) | 87 (1.1) |
| South Africa | 10-16 | 1271 | 89 (7.0) | 52 (4.1) | 661 (52.0) | 48 (3.8) | 105 (8.3) | 7 (0.6) |
| Spain | 6-13 | 631 | 59 (9.4) | 23 (3.7) | 57 (9.0) | 17 (2.7) | 21 (3.3) | 2 (0.3) |
| UK | 10-11 | 783 | 19 (2.4) | 44 (5.6) | 29 (3.7) | 0 | 5 (0.6) | 1 (0.1) |
| USA | 12-17 | 3114 | 80 (2.6) | 222 (7.1) | 525 (16.9) | 376 (12.1) | 201 (6.5) | 34 (1.1) |
| Total | 6-18 | 24605 | 1185 (4.8) | 1984 (8.1) | 6897 (28.0) | 1960 (8.0) | 1921 (7.8) | 270 (1.0) |
| ***NCEP criteria*** | | | | | | | | |
| Brazil | 15-17 | 441 | 101 (22.9) | 76 (17.2) | 181 (41.0) | 0 | 74 (16.8) | 17 (3.9) |
| China | 6-11 | 1416 | 416 (29.4) | 214 (15.1) | 28 (2.0) | 25 (1.8) | 115 (8.1) | 6 (0.4) |
| Greece | 8-17 | 439 | 184 (41.9) | 71 (16.2) | 41 (9.3) | 9 (2.1) | 65 (14.8) | 10 (2.3) |
| Iran | 6-18 | 8171 | 1712 (21.0) | 2075 (25.4) | 3056 (37.4) | 147 (1.8) | 1688 (20.7) | 234 (2.9) |
| Italy | 10-13 | 570 | 95 (16.7) | 69 (12.1) | 88 (15.4) | 1 (0.2) | 44 (7.7) | 5 (0.9) |
| Korea | 10-18 | 7769 | 1215 (15.6) | 1788 (23.0) | 1378 (17.7) | 134 (1.7) | 1032 (13.3) | 188 (2.4) |
| South Africa | 10-16 | 1271 | 289 (22.7) | 121 (9.5) | 632 (49.7) | 14 (1.1) | 210 (16.5) | 17 (1.3) |
| Spain | 6-13 | 631 | 229 (36.3) | 55 (8.7) | 57 (9.0) | 0 | 58 (9.2) | 12 (1.9) |
| UK | 10-11 | 783 | 181 (23.1) | 112 (14.3) | 29 (3.7) | 0 | 30 (3.8) | 4 (0.5) |
| USA | 12-17 | 3114 | 497 (16.0) | 544 (17.5) | 376 (12.1) | 51 (1.6) | 316 (10.1) | 57 (1.8) |
| Total | 6-18 | 24605 | 4918 (20.0) | 5125 (20.8) | 5866 (23.8) | 381 (1.5) | 3631 (14.8) | 550 (2.2) |

*Abbreviations BP* blood pressure, *FBG* fasting blood glucose, *HDL* high-density lipoprotein, *IDF* International Diabetes Federation, *NCEP* National Cholesterol Education Program, *TG* triglycerides, *UK* United Kingdom, U*SA* United States of America.

**Table S4** Comparison of proportion of children and adolescents among different subsamples from ten countries with ≥2 cardiometabolic risk factors based on IDF and NCEP criteria

| Country | Age  (years) | **Subsample 1**: all population, N (n, %) | **Subsample 2**: normal weight, N (n, %) | **Subsample 3**: normal weight plus thinness grade 1 and overweight, N (n, %) | **Subsample 4**: normal weight plus overweight and obesity, N (n, %) | **Subsample 5**: normal weight plus overweight, N (n, %) | **Subsample 6**: normal weight plus overweight and obesity and morbid obesity, N (n, %) |
| --- | --- | --- | --- | --- | --- | --- | --- |
| ***IDF criteria*** | | | | | | | |
| Brazil | 15-17 | 441 (32, 7.3) | 306 (12, 3.9) | 417 (28, 6.7) | 375 (28, 7.5) | 365 (25, 6.9) | 378 (28, 7.4) |
| China | 6-11 | 1416 (25, 1.8) | 849 (6, 0.7) | 1234 (17, 1.4) | 1291 (23, 1.8) | 1163 (17, 1.5) | 1336 (25, 1.9) |
| Greece | 8-17 | 439 (39, 8.9) | 225 (9, 4.0) | 378 (26, 6.9) | 384 (32, 8.3) | 347 (25, 7.2) | 398 (38, 9.6) |
| Iran | 6-18 | 8171 (849, 10.4) | 5014 (488, 9.7) | 7181 (724, 10.1) | 6151 (716, 11.6) | 5915 (654, 11.1) | 6199 (728, 11.7) |
| Italy | 10-13 | 570 (16, 2.8) | 290 (2, 0.7) | 492 (11, 2.2) | 523 (15, 2.9) | 466 (10, 2.2) | 538 (15, 2.8) |
| Korea | 10-18 | 7769 (628, 8.1) | 5285 (287, 5.4) | 7315 (530, 7.3) | 7012 (593, 8.5) | 6744 (511, 7.6) | 7043 (604, 8.6) |
| South Africa | 10-16 | 1271 (105, 8.3) | 864 (48, 5.6) | 1168 (89, 7.6) | 1129 (94, 8.3) | 1063 (83, 7.8) | 1154 (98, 8.5) |
| Spain | 6-13 | 631 (21, 3.3) | 242 (0, -) | 408 (5, 1.2) | 525 (16, 3.1) | 379 (5, 1.3) | 594 (21, 3.5) |
| UK | 10-11 | 783 (5, 0.6) | 509 (0, -) | 730 (2, 0.3) | 719 (5, 0.7) | 681 (2, 0.3) | 722 (5, 0.7) |
| USA | 12-17 | 3114 (201, 6.5) | 1792 (54, 3.0) | 2581 (114, 4.4) | 2774 (156, 5.6) | 2468 (110, 4.5) | 2974 (197, 6.6) |
| Total | 6-18 | 24605 (1921, 7.8) | 15376 (906, 5.9) | 21904 (1546, 7.1) | 20883 (1678, 8.0) | 19591 (1442, 7.4) | 21336 (1759, 8.2) |
| ***NCEP criteria*** | | | | | | | |
| Brazil | 15-17 | 441 (74, 16.8) | 306 (43, 14.1) | 417 (67, 16.1) | 375 (65, 17.3) | 365 (60, 16.4) | 378 (67, 17.7) |
| China | 6-11 | 1416 (115, 8.1) | 849 (36, 4.2) | 1234 (75, 6.1) | 1291 (104, 8.1) | 1163 (75, 6.5) | 1336 (115, 8.6) |
| Greece | 8-17 | 439 (65, 14.8) | 225 (16, 7.1) | 378 (45, 11.9) | 384 (58, 15.1) | 347 (45, 13.0) | 398 (65, 16.3) |
| Iran | 6-18 | 8171 (1687, 20.7) | 5014 (987, 19.7) | 7181 (1477, 20.6) | 6151 (1397, 22.7) | 5915 (1306, 22.1) | 6199 (1420, 22.9) |
| Italy | 10-13 | 570 (44, 7.7) | 290 (9, 3.1) | 492 (26, 5.3) | 523 (39, 7.5) | 466 (24, 5.2) | 538 (42, 7.8) |
| Korea | 10-18 | 7769 (1032, 13.3) | 5285 (490, 9.3) | 7315 (874, 12.0) | 7012 (978, 14.0) | 6744 (847, 12.6) | 7043 (994, 14.1) |
| South Africa | 10-16 | 1271 (210, 16.5) | 864 (117, 13.5) | 1168 (176, 15.1) | 1129 (195, 17.3) | 1063 (170, 16.0) | 1154 (203, 17.6) |
| Spain | 6-13 | 631 (58, 9.2) | 242 (6, 2.5) | 408 (14, 3.4) | 525 (42, 8.0) | 379 (14, 3.7) | 594 (58, 9.8) |
| UK | 10-11 | 783 (30, 3.8) | 509 (11, 2.2) | 730 (21, 2.9) | 719 (29, 4.0) | 681 (21, 3.1) | 722 (30, 4.2) |
| USA | 12-17 | 3114 (316, 10.2) | 1792 (76, 4.2) | 2581 (170, 6.6) | 2774 (241, 8.7) | 2468 (166, 6.7) | 2974 (311, 10.5) |
| Total | 6-18 | 24605 (3631, 14.8) | 15376 (1791, 11.6) | 21904 (2945, 13.4) | 20883 (3148, 15.1) | 19591 (2728, 13.9) | 21336 (3305, 15.5) |

*Abbreviations IDF* International Diabetes Federation, *NCEP* National Cholesterol Education Program, *UK* United Kingdom, *USA* United States of America.

**Table S5** Results from ROC curve analyses to identify relatively optimal cut-offs of WHtR to discriminate those with ≥2 cardiometabolic risk factors from ten countries using the second analysis strategy

| Country | Age  (years) | Sample size | IDF criteria | | | |  | NCEP criteria | | | |
| --- | --- | --- | --- | --- | --- | --- | --- | --- | --- | --- | --- |
|  |  |  | Optimal cut-offs | AUC (95% CI) | Sensitivity | Specificity |  | Optimal cut-offs | AUC (95% CI) | Sensitivity | Specificity |
| Brazil | 15-17 | 441 | 0.44 | 0.699 (0.599-0.800) | 0.719 | 0.680 |  | 0.45 | 0.591 (0.513-0.669) | 0.419 | 0.763 |
| China | 6-11 | 1416 | 0.48 | 0.766 (0.682-0.850) | 0.840 | 0.692 |  | 0.47 | 0.661 (0.605-0.717) | 0.661 | 0.661 |
| Greece | 8-17 | 439 | 0.49 | 0.654 (0.569-0.739) | 0.821 | 0.488 |  | 0.49 | 0.662 (0.589-0.734) | 0.815 | 0.508 |
| Iran | 6-18 | 18557 | 0.44 | 0.592 (0.570-0.615) | 0.571 | 0.613 |  | 0.46 | 0.578 (0.561-0.595) | 0.393 | 0.763 |
| Italy | 10-13 | 570 | 0.51 | 0.692 (0.576-0.809) | 0.812 | 0.572 |  | 0.51 | 0.669 (0.584-0.754) | 0.750 | 0.587 |
| Korea | 10-18 | 13352 | 0.45 | 0.677 (0.653-0.701) | 0.639 | 0.716 |  | 0.44 | 0.661 (0.641-0.681) | 0.650 | 0.672 |
| South Africa | 10-16 | 1271 | 0.46 | 0.635 (0.575-0.695) | 0.514 | 0.756 |  | 0.45 | 0.607 (0.559-0.654) | 0.505 | 0.709 |
| Spain | 6-13 | 631 | 0.51 | 0.751 (0.669-0.833) | 0.905 | 0.597 |  | 0.52 | 0.766 (0.702-0.830) | 0.862 | 0.670 |
| UK | 10-11 | 783 | 0.50 | 0.894 (0.880-0.908) | 1 | 0.788 |  | 0.49 | 0.658 (0.554-0.762) | 0.567 | 0.749 |
| USA | 12-17 | 3114 | 0.49 | 0.682 (0.642-0.721) | 0.721 | 0.642 |  | 0.49 | 0.694 (0.661-0.728) | 0.731 | 0.658 |

Abbreviations: AUC = area under the curve; CI = confidence interval; IDF = International Diabetes Federation; NCEP = National Cholesterol Education Program; ROC = receiver operating characteristic; UK = United Kingdom; USA = United States of America; WHtR = waist-to-height ratio.

**Table S6** Results from ROC curve analyses to identify relatively optimal cut-offs of WHtR to discriminate those with ≥3 cardiometabolic risk factors from ten countries

| Region | Age  (years) | Sample size | IDF criteria | | | |  | NCEP criteria | | | |
| --- | --- | --- | --- | --- | --- | --- | --- | --- | --- | --- | --- |
|  |  |  | Optimal cut-offs | AUC (95% CI) | Sensitivity | Specificity |  | Optimal cut-offs | AUC (95% CI) | Sensitivity | Specificity |
| ***Europe and USA*** | 6-17 | 5537 | 0.50 | 0.735 (0.673-0.798) | 0.833 | 0.637 |  | 0.52 | 0.769 (0.723-0.816) | 0.818 | 0.720 |
| *Europe* | 6-17 | 2423 | 0.49 | 0.781 (0.771-0.791) | 1 | 0.561 |  | 0.51 | 0.762 (0.694-0.831) | 0.871 | 0.653 |
| *USA* | 12-17 | 3114 | 0.51 | 0.765 (0.693-0.837) | 0.824 | 0.707 |  | 0.52 | 0.773 (0.714-0.832) | 0.807 | 0.739 |
| ***Asia, Africa, and South America*** | 6-18 | 19068 | 0.46 | 0.716 (0.681-0.751) | 0.705 | 0.727 |  | 0.46 | 0.686 (0.661-0.711) | 0.641 | 0.732 |
| *Asia* | 6-18 | 17356 | 0.46 | 0.715 (0.679-0.752) | 0.705 | 0.726 |  | 0.46 | 0.685 (0.659-0.711) | 0.640 | 0.730 |
| *Africa* | 10-16 | 1271 | 0.46 | 0.653 (0.458-0.849) | 0.571 | 0.735 |  | 0.46 | 0.723 (0.602-0.843) | 0.706 | 0.739 |
| *South America* | 15-17 | 441 | 0.47 | 0.914 (0.897-0.932) | 1 | 0.829 |  | 0.45 | 0.697 (0.563-0.832) | 0.647 | 0.748 |

Abbreviations: AUC = area under the curve; CI = confidence interval; IDF = International Diabetes Federation; NCEP = National Cholesterol Education Program; ROC = receiver operating characteristic; WHtR = waist-to-height ratio.

**Table S7** Results from ROC curve analyses to identify relatively optimal cut-offs of WHtR to discriminate those with ≥2 cardiometabolic risk factors in external independent test pediatric populations from six countries

| Country | Age  (years) | Sample size | IDF criteria | | | |  | NCEP criteria | | | |
| --- | --- | --- | --- | --- | --- | --- | --- | --- | --- | --- | --- |
|  |  |  | Optimal cut-offs | AUC (95% CI) | Sensitivity | Specificity |  | Optimal cut-offs | AUC (95% CI) | Sensitivity | Specificity |
| Brazil | 6-18 | 824 | 0.46 | 0.686 (0.564-0.808) | 0.600 | 0.772 |  | 0.47 | 0.636 (0.559-0.713) | 0.458 | 0.814 |
| China | 7-17 | 749 | 0.46 | 0.656 (0.566-0.746) | 0.535 | 0.778 |  | 0.46 | 0.578 (0.509-0.647) | 0.405 | 0.721 |
| Germany | 6-18 | 6810 | 0.49 | 0.593 (0.563-0.624) | 0.404 | 0.831 |  | 0.49 | 0.600 (0.574-0.627) | 0.418 | 0.784 |
| Italy | 6-18 | 724 | 0.52 | 0.632 (0.573-0.691) | 0.875 | 0.463 |  | 0.52 | 0.635 (0.586-0.684) | 0.841 | 0.489 |
| Korea | 12-15 | 152 | 0.46 | 0.667 (0.550-0.784) | 0.815 | 0.576 |  | 0.47 | 0.681 (0.576-0.785) | 0.768 | 0.594 |
| USA | 12-18 | 360 | 0.51 | 0.656 (0.562-0.750) | 0.660 | 0.652 |  | 0.51 | 0.650 (0.549-0.752) | 0.658 | 0.643 |

Abbreviations: AUC = area under the curve; CI = confidence interval; IDF = International Diabetes Federation; NCEP = National Cholesterol Education Program; ROC = receiver operating characteristic; USA = United States of America; WHtR = waist-to-height ratio.
